# Determination of phosphorus compounds in plant tissues: from colourimetry to advanced instrumental analytical chemistry

**DOI:** 10.1186/s13007-022-00854-6

**Published:** 2022-02-21

**Authors:** Dorota Wieczorek, Beata Żyszka-Haberecht, Anna Kafka, Jacek Lipok

**Affiliations:** Department of Pharmacy and Ecological Chemistry, Institute of Chemistry, University of Opole, Oleska 48, 45-052 Opole, Poland

**Keywords:** Phosphorus, Plant tissues, Determination, Analytical methods, NMR

## Abstract

Although the spectrum of effective methods and techniques that allow determination of inorganic or total phosphorus is impressive, more precise analysis of these substances in plant tissues is not a routine or trivial task. The complexity of chemical composition of plant tissues treated as the analytical matrices is thought to be the main cause why there is no one answer, how appropriate phosphorus compounds may be determined qualitatively and quantitatively. Even if more advanced spectrophotometric measurements and classical variants of absorption (FAAS) or emission (ICP-AES/ ICP-OES) spectrometry techniques are used, it is necessary at first to isolate various forms of phosphorus from the matrix, and then to mineralize them prior the determination. Significant progress in such a kind of analytical efforts was brought by implementation of combined methods e.g. ETV-ICP-AES or HR-ETAAS, does allow the isolation of the phosphorus analyte and its detection during a kind of “one step” analytical procedure, directly in plant tissues. Similar benefits, regarding sensitivity of determinations, are obtained when XRF, SIMS or nanoSIMS—more expensive techniques of imaging the presence of phosphorus in biological matrices have been used. Nowadays, obviously being aware of higher limit of detection, nuclear magnetic resonance spectroscopy, especially the ^31^P NMR technique, is thought to be the most universal analytical tool allowing to determine various chemical forms of plant phosphorus qualitatively and quantitatively, at the same time. Although ^31^P NMR provides valuable information about the phosphorus profile of plants, it should be emphasized that each analytical issue related to the determination of phosphorus compounds in plant tissues and organs, requires an individual approach to defined problem.

## Background

Plants require at least sixteen nutritional elements for normal growth and completion of their life cycle. Among these elements are substances referred to as macronutrients and micronutrients. Macronutrients are required by plants in large quantities (in concentrations exceeding 1 ppm or 1–150 g per kg plant dry matter) and include elements such as carbon, hydrogen, oxygen, nitrogen, phosphorus, potassium, calcium, magnesium, and sulphur. Regarding micronutrients, among which iron, manganese, zinc, and copper are reported the most often, plants require lower concentrations below 1 ppm (or 0.1–100 mg per kg plant dry matter) [1]. Although all nutrients are extremely important for proper physiological and biochemical conditions in plants, phosphorus (P), a nonmetallic chemical element, seems to play a crucial role, especially in light of limitations of crop production in most regions of the world [2]. Plants obtain phosphorus from the soil in the forms of H_2_PO_4_^−^ and HPO_4_^2−^ [3]. Although the total phosphorus content in soil can be relatively high, the acquisition of this element by plants is often limited because inorganic phosphates are characterized by low solubility and a high sorption capacity in soil [4]. Some important soil properties that influence the solubility of phosphorus compounds are pH, the concentrations of iron, aluminium, calcium ions and the nature of the soil particles, mainly their surface area [2]. Independent of the mentioned limitations and the different needs of individual plant species, the phosphorus concentration in plants ranges from 0.05 to 0.5% of plant dry weight [5]. This element occurs in plants either as the free inorganic orthophosphate group (Pi) or as organophosphorus compounds [6]. Pi existing in plant cells is divided into two physiologically different pools. The first is located in the cytoplasm and constitutes the metabolically active Pi pool, while the second, which is a specific phosphorus reserve, is stored in the vacuoles [6]. Under a sufficient P supply, 85–95% of the cellular P is found in the vacuole, and only 5–15% constitutes the cytoplasmic pool [7].

Organophosphorus compounds occurring in living organisms are mostly organic phosphate esters, in which phosphate groups are bound to organic units by a C-O-P linkage. The main pools for esterified P are nucleic acids (DNA and RNA), phosphoproteins, phospholipids, sugar phosphates, and energy-rich phosphate compounds (e.g., adenosine triphosphate) [8]. Phospholipids are a class of lipids whose structure is based on the glycerol backbone to which two long chains of fatty acid acyl groups and orthophosphoric acid residues are attached. The base phospholipid class is phosphatidic acids with one phosphate group bound to the third carbon atom of glycerol. Modification of the phosphate group by the addition of choline, serine, ethanoloamine, inositol or glycerol leads to the formation of structurally different classes of phospholipids referred successively to as phosphatidylcholine, phosphatidylserine, phosphatidylethanolamine, phosphatidylinositol or phosphatidylglycerol [9]. Phospholipids possess many vital functions in plant cells. These substances are not only the most important building components of cell membranes but also participate in membrane trafficking, cytoskeletal arrangement, and signal transduction [10]. By changing the physical properties of membranes, they increase or decrease the activity of transport proteins (i.e., membrane ATPase) and thereby help regulate membrane transport. Phospholipid metabolism plays a crucial role in embryo maturation and seed germination, and participates also in proper pollen development, cotyledon vein vascular development, stress responses, and signal transduction (light and sugar) [11].

Another important group of phosphate esters is sugar phosphates. Phosphate derivatives of sugars are generally essential metabolic intermediates, and some of them, such as phytic acid (Ins P6), are also a reservoir of phosphorus in plants. For example, trehalose-6-phosphate (T6P) is a critical signalling metabolite involved in regulating plant growth and development in response to carbon availability [12]. Chemically, phytic acid is a sixfold dihydrogen phosphate ester of myo-inositol. In seeds, it comes as a mixed salt of several cations, including potassium, magnesium, calcium, manganese, iron, and zinc, and accumulates in membrane-bound inclusions referred to as globoids [13]. Depending on the plant species, it is estimated that the content of phosphorus derived from phytate ranges from 30 to 60% of the total phosphorus in seeds. It is generally assumed that the major role of Ins P6 in plants is to act as a storage form for Pi, but these compounds also have other physiological roles other than P6 storage of phosphorus, such as mRNA export, chromatin remodelling, and DNA double-strain repair. The ability of phytic acid to complex iron ions (Fe^2+^) contributes to reducing the formation of reactive oxygen species during the lipid peroxidation reaction [14].

There are various forms of phosphate derivatives of nucleotides containing up to three esterified phosphate residues associated with ribose or deoxyribose units. Phosphodiester bonds connecting repeating nucleosides form an important part of the structural skeleton of ribonucleic and deoxyribonucleic acids. A large group of phosphoester nucleoside derivatives (e.g., ATP, ADP, UDP) is a specific primary reservoir of energy distributed in phosphorylation and dephosphorylation reactions of other chemical compounds. The same substances are also substrates of exoenergetic hydrolysis reactions of phosphoester bonds due to which energy used in endoenergetic metabolic transformations appears in biological systems. The role of ATP as an energy source is well known, but ATP also plays a different role in the extracellular matrix. Extracellular ATP (eATP) in plants is a specific signalling agent that generates an increase in cytosolic Ca^2+^, contributing to plant growth and defence responses [15]. Other cytosolic derivatives of nucleoside phosphate compounds take part in signal transduction processes (including cyclic AMP [cAMP]), ion transport and channel regulation, plant defences, activation of enzymes and phytochrome action.

**Fig. 1:**
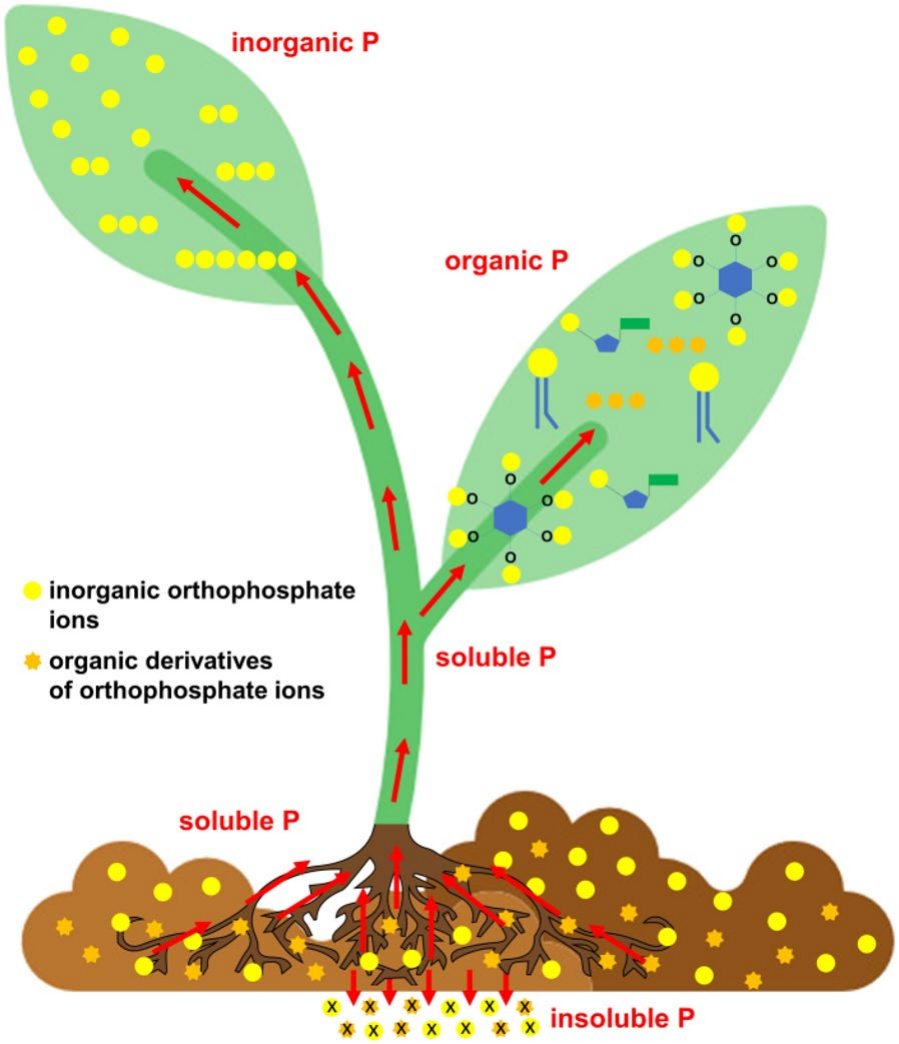
Diagram of the occurrence and translocation of various forms of phosphorus in soil and plant

The variety of the forms of phosphorus compounds occurring in plant cells and tissues, together with the complexity of plant tissues as analytical matrices (Fig. 1), are the reasons why there is no one best answer regarding how those substances may be determined qualitatively and quantitatively. Therefore, in this paper, we try to suggest how it can be done depending on the examined forms of phosphorus and the purpose of the determination.

## Overview of the methods used in the determination of phosphorus in plants

### Analysis of phosphorus in plant material

Many analytical techniques have been employed to characterize forms of phosphorus in plant material. Among them, spectrophotometric methods based on molybdenum blue colouration are still the most widely used. However, colourimetric methods may not be sufficient to characterize different species of organic and inorganic P. To respond to these challenges, in recent years, many spectroscopic, chromatographic, and electrophoretic techniques have been developed and successfully used by workers [16].

#### Determination of the total phosphorus content (TP) in plants

The most commonly used measure of the amount of phosphorus in plants is the total phosphorus content (TP). TP is the sum of the contents of all P-containing compounds in the plant sample. In plant tissues, phosphorus occurs in organic forms such as phosphate monoester (C-O-P bond) (e.g., phytate), phosphate diesters (C–O–P–O–C bond) (e.g., nucleotides and phospholipids), and inorganic forms such as orthophosphate, pyrophosphate (P-O-P bond), and polyphosphate. The level of TP in plants has been reported in many studies, and it varies within the range of mg/g of dry plant tissue. Moreover, it was found that approximately 75% (± 10%) of the TP in plant material, specifically in mature seeds, is present as phytic acid or phytate, whereas orthophosphate is the dominant form of P in other parts of the plant (root, stem, leaf, chaff/pod) [17].

TP can be determined by various spectrophotometric (molybdenum blue colourimetric method, malachite green assay) or spectrometric methods (FAAS, ETAAS, ICP-OES, ICP-AES or ICP-MS) [18] (Table 1). However, regardless of the method used for the determination of TP, the analysis of the tested material has to be preceded by the process of mineralization. During mineralization carried out, e.g., by chemical oxidation or heating in microwave ovens with the addition of strong acids, organic substances are decomposed to simple inorganic compounds. Unfortunately, in this regard, the accuracy of all mentioned methods depends on the effectiveness of the total destruction of the plant material because incomplete solubilization of the sample can lead to an understated content of phosphorus. Moreover, the digestion of plant material is time-consuming and dangerous, requiring concentrated acids at high temperature.

**Table 1:** Summary of analytical methods, their advantages and limits used for the determination of total phosphorus in plants

Method	Advantages	Disadvantages
Colorimetric assays		
Molybdenum blue method	Higher range of linear response (up to ~ 13 μm Pi) than malachite green assay (up to ~ 6 μm Pi) Automation possible	Complete destruction of the sample Discrimination between the different pools of phosphate (e.g. orthophosphate, hydrogen phosphate) impossible
Malachite green method	Easy to implement Stability of reagents Five times more sensitive than the molybdenum blue method - downscaling possible (range of detection 0.3 to 8 ng of Pi)	
ICP-AES and ICP-MS	Quantification of P and other elements in a single analysis	Complete destruction of the sample Discrimination between various P-metabolites impossible
FAAS/ HR-CS FAAS	Low operational costs Good analytical performance HR-CS FAAS improves sensitivity and detectability	Complete destruction of the sample Limited sensitivity, its capability to only measure one element at a time and limited linearity
HR-CS ETAAS	High sample throughput, good sensitivity, sufficient precision, and straightforward calibration with aqueous standards	Complete destruction of the sample Single-element analysis
X-ray spectrometry (XRF)	High sensitivity Compatible with measurements at ambient temperature and pressure	Relative quantification Discrimination between various P-metabolites impossible Difficult sample preparation
Secondary Ion Mass Spectrometry (SIMS)	High sensitivity P imaging in the different compartments of the cell Possibility to colocalize P with other elements Discrimination between isotopes	Relative quantification Discrimination between various P-metabolites impossible Difficult sample preparation

In recent years, research efforts devoted to quantifying phosphorus in plants have moved from colourimetry to ICP spectrometry. The main reason is the increasingly critical attitude to the spectrophotometric measurement due to the significant interference problems upon P determination, limited stability of reducing agents and slow rate for colour formation [16]. ICP techniques are based on the excitation of atoms and ions with plasma torch and analysis of the emitted wavelength of electromagnetic radiation, characteristic of a particular element ICP-AES (also called ICP-OES). Emitted radiation can be measured both qualitatively (in terms of wavelength) and quantitatively (in terms of intensity) [19]. Alternatively, it is possible to detect elements by a mass spectrometer using ICP-MS. In this case, the identification of the elements is based on their mass-to-charge ratio.

ICP-OES showed great potential as an alternative, instrumental method to the current officially accepted colourimetric method of molybdenum blue. The ICP-OES method is relatively simple, time-consuming and has good linearity. Moreover, it provides the opportunity to simultaneously determine the macroelements (exceptions are elements such as hydrogen, carbon, nitrogen, and oxygen that are lost during the mineralization process) and microelements contained in plants. The main advantage of ICP-OES is its low limit of detection (LOD); hence, the application of this method allows the determination of even trace amounts of phosphorus (at the level of μg L^−1^) in plant materials. The detection limit of these techniques can vary depending on the presence of other substances in the sample. Increasing the plasma temperature can improve ionization and sensitivity for P, but in exchange, this also increases the background level [16, 19]. Importantly, the determination of P in plants by ICP-OES analysis requires prior dissolution of plant samples through acid digestion [20]. However, Masson (2011) proposed direct phosphorus analysis on solid plant samples by electrothermal vapourization (ETV) coupled with ICP-AES to overcome these analytical difficulties. The application of the ETV technique for the determination of P in plant samples enables the avoidance of matrix interference and offers a convenient analysis of small plant samples. ETV-ICP-AES is a fast, inexpensive, and reliable instrumental technique, and accurate quantification can be accomplished easily by using standard aqueous solutions. The ICP-MS method is used less frequently in the determination of TP in plants, although it overcomes the sensitivity limitations of ICP-OES [21].

Another atomic spectrometric technique useful for the determination of phosphorus in plants is line source flame atomic absorption spectrometry (LS-FAAS). However, because of limitations of AAS concerning accuracy and sensitivity, single-element analysis and narrow-range calibration and emission techniques (ICPs) are more frequently chosen by scientists to determine phosphorus in plant tissues [22].

Additionally, the use of electrothermal atomic absorption spectrometry (ETAAS), and more specifically high resolution-continuum source ET spectrometry, thanks to the judicious selection of the chemical modifier and the temperature program, permits the direct determination of phosphorus in plant tissues by monitoring both atomic and molecular absorption spectrometry in the vicinity of the 213.618 nm line. In both cases, the advantages of these methods are the high sample throughput, good sensitivity, sufficient precision, and straightforward calibration with aqueous standards [23].

Given the mentioned typical problems of the described atomic spectroscopy techniques, the use of other methods to image P in plant tissues and cells has increased over the last few years. Among these methods worth mentioning are X-ray fluorescence spectrometry (XRF) and secondary ion mass spectrometry (SIMS). Both of these techniques are based on elemental analysis of the surface of a sample bombarded by photons or ion beams. XRF and SIMS offer several advantages, such as a high resolution (subcellular level), the possibility to image several elements at one measurement and the possibility to discriminate between different isotopes. XRF works on the principle of excitation of inner orbital electrons by an X-ray radiation source. When the excited electrons return to the ground state, they fluoresce, which means that they release photons of energy and wavelength characteristic of the given atoms [24]. The penetrating nature of X-rays also allows the investigation of the internal distribution of elements in dry or dehydrated tissues, including applications such as 3D tomography on seeds [25]. Future technological developments of X-ray methods could enable the separate differentiation of the various P forms in plant samples. The suitability of XRF instruments over conventional spectroscopic techniques for determining the elemental composition of plants, including P, has been demonstrated in several studies [26, 27]. XRF provides a fast, safe, nondestructive and potentially more precise method to determine the P content in plant matrices. Despite the undeniable advantages, XRF is not routinely used by scientists for the elemental analysis of plants. The most important reason for this is very expensive XRF instrumentation compared to the equipment typically used in digestion-based elemental analysis techniques. Furthermore, many XRF analysers require relatively high amounts of plant material (approximately 1 to 10 g) for analysis [24].

Secondary ion mass spectrometry (SIMS) is based on the emission and analysis of secondary particles resulting from the bombardment of the surface of the sample by energetic primary ions, in the case of P analysis, a Cs^+^ beam, in a vacuum. During this process, the top few atomic layers of the sample surface are sputtered, and the emitted secondary particles are analysed with a mass spectrometer to determine the chemical distribution of the sample. The possibility of detecting multiple ionic species from the same analysed area allows relative elemental quantification, including ^12^C, ^14^ N, ^32^S, and ^31^P, but cannot distinguish inorganic P among detected forms of phosphorus. Nanoscale secondary ion mass spectrometry (NanoSIMS), a recent development in SIMS that combines high sensitivity with high spatial resolution, despite drawbacks such as the requirement for high vacuum, allows us to show that P is localized in plant cells in the form of free inorganic P, DNA, RNA, lipids and proteins in a large range of plant tissues, including grains [28], leaves [29] and roots [30, 31].

#### Determination of inorganic phosphorus in plants

**Fig. 2:**
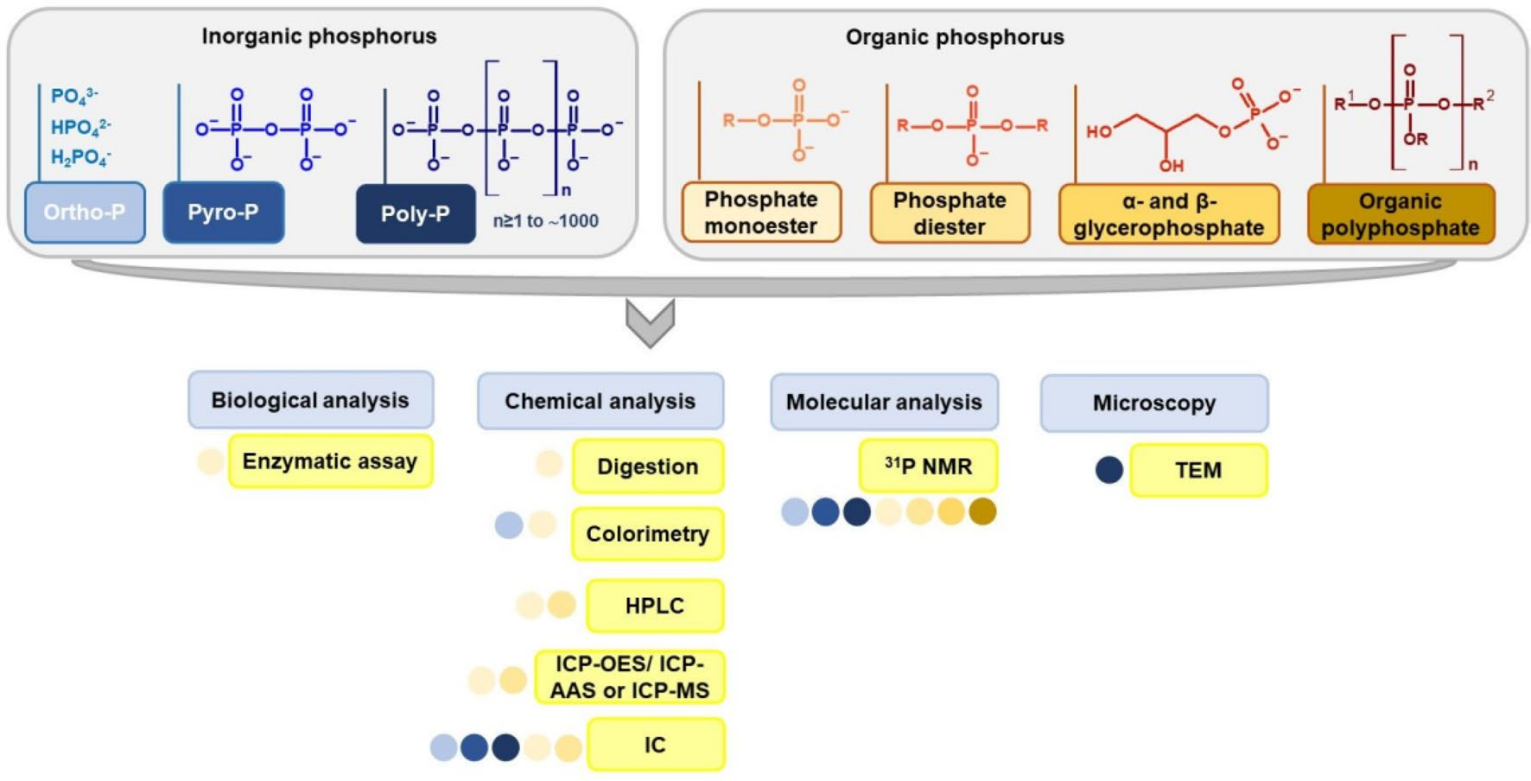
Inorganic and organic forms of phosphorus present in plants forming the total phosphorous pool together with an indication of the methods allowing for determination of particular forms of P in plant material

The fraction of inorganic phosphorus (inorganic P, Pi) occurs in plants as approximately half of the total phosphorus (TP) and mainly as orthophosphates, which can be present as H_3_PO_4_, H_2_PO_4_^−^, HPO_4_^2−^ and PO_4_^3−^, with respective pKa values of 2.12, 7.21, and 12.67. Among those forms, H_2_PO_4_^−^ is the most efficiently absorbed by plants [32, 33]. The remaining forms of inorganic phosphorus present in plants are pyrophosphates [34] and polyphosphates [35] (Fig. 2).

Most methods for phosphate determination are based on the spectrophotometric detection of a coloured phosphomolybdate complex [36]. Two colourimetric methods, the molybdenum blue method [37] and the malachite green assay [38], are commonly used to quantify orthophosphate extracted from plants. The indication for molybdenum blue takes place in two stages. The first stage involves the reaction between orthophosphate ions and molybdate ions in an acidic solution and results in the formation of a yellow phosphomolybdate complex. In contrast, the second stage entails reducing molybdenum in the complex to form an intense blue-coloured product.

All molybdenum blue methods are normally applied in an aqueous solution and require a strong acid, a source of molybdate (Mo(VI)) and a reductant. Ascorbic acid is the most widely used reductant, and antimony is used to catalyse the reduction of molybdenum by ascorbic acid. The absorbance of the resulting complex was measured with a spectrophotometer at 880 nm [39].

One of the most sensitive methods for the determination of phosphate is the colourimetric method with malachite green, also called the micromethod, because it allows the determination of even nanomolar concentrations of phosphorus in samples. Aromatic amine called malachite green, in the presence of ammonium molybdate reacts stoichiometrically with Pi and forms a coloured complex characterized by a maximum absorption at 660 nm [40]. Any colourimetric method detects free phosphate, although it cannot distinguish between the different pools of P present in the sample [41, 42]. Both spectrophotometric methods, molybdenum blue and malachite green, have their pros and cons [42]. The molybdenum blue method can be automated by flow injection analysis [43] and has a broad calibration range of linearity at high and very low concentrations (0.004–1.2 mg Pi L^−1^). The appearance of colour in this method is independent of temperature, but the method can be time-consuming, labour-intensive, and may generate significant chemical waste, as some reagents are briefly stable at room temperature; thus, they have to be replaced with newly prepared reagents [44]. For comparison, the method with malachite green is linear over a range between 0.007–0.6 mg Pi L^–1^ [42] and is widely used in plant science research due to its simplicity and the high stability of the assay reagents [19]. In both procedures, the adaptation of the assay to small samples may allow accurate measurements of Pi in the range of nanograms (of 0,3 to 8 ng of Pi per sample). The calibration curve and the sample volume are crucial elements for the reproducibility and accuracy of results. The disadvantages of both mentioned methods are the destruction of plant tissues' integrity and the limitation of measurements to the total content of Pi in plant samples. Nevertheless, the sensitivity of those methods and their reliability make it feasible to analyse small samples at the tissue level [19].

Ion chromatography (IC), a subset of liquid chromatography, is the most frequently chosen chromatographic method for the determination of phosphates. This method was first elaborated to measure the concentrations of inorganic anions with a conductivity detector [45]. In comparison to colourimetric methods, IC allows the detection of orthophosphate ions in real-time and synchronous analysis of ortho- and pyrophosphate anions and other ionic species [46]. The analyses of environmental samples showed that the concentration of Pi determined by the IC method was usually less than that obtained by the molybdenum blue method [47]. Many IC methods for routine anion determination, including inorganic phosphate, have been well documented as standard methods [48]. When using the IC technique, the stationary phase and eluent should be matched to the type of samples. It should also be noted that especially a high ion content may influence the obtained results. The presence of high concentrations of anions (chloride, nitrate, and sulphate) may disrupt or even make the IC determination of phosphate impossible [49]. Depending on the type of cations, the peak areas of phosphate may fluctuate. It was proven that ferric iron(III) significantly decreases, whereas aluminium(III) ions slightly increase the peak areas during phosphate IC determination [50].

Despite various interferences in real samples, the analysis of phosphates by IC has been more accepted for complex environmental samples such as plant samples, especially when it is compared to the molybdenum blue method [51, 52].

Current scientific reports indicate that inorganic polyphosphates (polyP) may also exist in plants [53]. Polyphosphates are linear polymers of inorganic phosphate (Pi) units with sizes ranging from tripolyphosphate (three Pi units) to long-chain polyP (approximately 1000 Pi units) linked by phosphoanhydride bonds. The existence of polyP in plant tissues has been established using microscopy (TEM) [54, 55] and biochemical methods of extraction [56], which are now known to produce artefacts. Recently, polyP-specific dyes and polyP-binding domains were used to detect polyP in plant cells. Although the presence of significant polyP stores was not confirmed in plants, it is possible that higher plants accumulate polyP in specific organs or cells, only in certain developmental stages or in response to certain environmental stimuli [35].

#### Determination of organic forms of phosphorus in plants

Organic phosphorus (organic P, Po) occurs in plant tissues and cells as phosphate monoester (C-O-P bond) (e.g., phytate), phosphate diesters (C-O–P–O-C bond) (e.g., nucleotides and phospholipids), α- and β-glycerophosphates, and organic polyphosphates [57].

The phytate form of phosphorus consists of phytic acid (phytate; myo-inositol-1,2,3,4,5,6-hexakisphosphate, InsP6), which is the most abundant P-containing compound in plant seeds. Although phytates are not the one or even the crucial form of organic phosphorus in plant tissues, the vast majority of analytical reports are addressed to this fraction. Nevertheless, the determination of phytic acid in plants is still an analytical challenge because this compound does not have chromophore or fluorophore groups and is chemically stable, limiting its derivatization to be detected by spectrophotometric techniques without prior separation [57].

Typically, separated phytate is measured indirectly by displacing coloured metal complexes or by determining inorganic phosphate obtained by the enzymatic process of digestion of this compound [58]. Phytate phosphorus (PPhy) can be determined by the colourimetric method with the pink coloured Wade reagent (0.03% FeCl_3_ solution containing 0.3% sulfosalicylic acid solution, 3/1 (v/v)) in an acid medium with the formation of a complex that can be spectrophotometrically measured at 500 nm. In the presence of phytate, ferric ions bind preferentially to PPhy, thus resulting in a decrease in the intensity of the Wade reagent [59]. However, as Fe^3+^ does not discriminate between the PPhy or the Pi present in the various plant sources, it is necessary that, before spectrophotometric dosing, the sample after acidic or basic extraction is eluted by an anion resin column to separate PPhy from Pi [59, 60]. After correlation with a standard curve of phytic acid, the value obtained should be multiplied by 0.282 (molar ratio of P in the IP6 molecule) to express the content of Pphy in the plant sample [61]. This method seems to be time-consuming due to the anion exchange chromatographic (AEC) separation of phytates. In 2007, Gao and coworkers described the modified colourimetric (Wade reagent based) method to overcome poor precision and reproducibility [62]. The proposed changes include extending the extraction time to 16 h, lowering the centrifugation temperature to 10 °C and adding a matrix cleaning step to improve phytate recovery. Another most acceptable method is AOAC method 986.11 [63], which can quantify InsP6 via the determination of inorganic phosphorus by a spectrophotometric method after traditional extraction with an anion exchange column and acidic digestion. This method also has limitations; inter alia, each individual analysis of plant samples requires arduous, time-consuming AEC, and the content of InsP6 in some plants may be overestimated because it cannot distinguish between InsP3-InsP5, if present, and InsP6. Additionally, some plant components, such as nucleotides, can also give elevated values of InsP6 [64]. Quite recently, in 2016, a simple and sensitive spectrophotometric method for the determination of phytic acid in grains based on the formation of a coloured complex between glyoxal bis(2-hydroxianiline) and calcium ions in an alkaline medium was developed [65]. The selectivity of this method was evaluated for 15 possible interferences among anions and cations, and the results when compared with the reference procedure (Wade method), showed no significant difference [65].

The activity of phytases (myo-inositol hexakisphosphate 3- and 6-phosphonohydrolases; EC 3.1.3.8 and 3.1.3.26), the common plant enzymes that catalyse the phosphate monoester hydrolysis of phytate, results in the sequential formation of a series of lower phosphoric esters (myo-inositol pentakis-, tetrakis-, tris-, bis-, and monophosphates) and the release of inorganic phosphate [66, 67]. Carvalho Vieira and Nogueira proposed a simple, rapid, and automated flow injection enzyme-spectrophotometric procedure to determine phytate in plants. In this method, the phytate was hydrolysed by the enzyme phytase coupled to a solid phase packed into an enzymatic reactor, and the obtained hydrolysed orthophosphate was determined by a spectrophotometric method dedicated to the analysis of Pi [43]. Based on a similar idea, in 2016, McKei proposed acid extraction and determination of phytic acid and myo-inositol phosphates from various plant samples. In this case, the phosphate released from phytic acid via enzymatic dephosphorylation was measured using a modified colourimetric molybdenum blue assay and calculated as the TP or phytic acid content of the original sample. This relatively simple and high-throughput method has many advantages over other existing methods used to measure phytic acid. It does not require expensive equipment, nor does it involve time-consuming or throughput-limiting steps such as AEC [57].

The most widely disseminated direct analytical strategies to determine InsP6 or other InsPn in plants are based on numerous instrumental techniques, such as IC, liquid chromatography with various detection systems and atomic spectrometric techniques, such as ICP-OES and ICP-MS, associated or not with chromatography.

Ion pair chromatography (IPC) and high-performance ion chromatography (HPIC) are the most commonly used methods for determining InsP6 and other InsPn, most of which are capable of simultaneous separation and determination of these compounds [68]. Using the IPC procedures developed by [69], it is possible to separate and quantitatively determine InsP3 to InsP6 based only on the number of phosphate groups in the myo-inositol ring without differentiating isomeric forms of InsP6. HPIC addition allows the differentiation of the isomeric forms of InsPn with the same number of phosphate groups [70]. A serious limitation of the more common use of these techniques is the interference evoked by ATP and ADP occurring naturally in plant matrices.

Unfortunately, because not all of the InsPn isomers are commercially available, purity information is not provided for some of those standards. It is always an important challenge to identify all of the chromatographic peaks that have been separated in any particular study and accurately quantify individual InsPn isomers, even InsPn, since a certified reference material for InsPn, which can give the exact purity information, is not currently available. The estimation of InsP3-InsP5 can be conducted by using the InsP6 standard in combination with the correction factors or relative response factors of InsP3-InsP5 to InsP6. Because InsP3-InsP5 pure standards are needed in most methods, accurate purity information is usually lacking, even when using similar treatment methods, the results obtained in different studies are conflicting [68]. Gradient elution allows the separation and quantitation of InsP6–InsP1 and their positional isomers [71]. Chen and Li reported the procedure that involved HPIC with the acidic system and post-column UV detection, in which all 35 possible InsP2-InsP6 isomers (excluding enantiomers) were separated into 27 peaks and the elution order of all InsP2-InsP6 isomers was definitively established [72]. The same author proposed a high-performance anion-exchange chromatographic method (HPAEC), in which an average relative response factor of penta- to hexasphosphates was determined by further research on InsP6 hydrolysis, and accurate analysis of InsP6 purity was also carried out. This method has been successfully applied to the determination of phytic acid and inositol pentakisphosphates in various plant samples. In 2005, a useful method for the analysis of phosphorus compounds focused on sugar phosphates from the model higher plant *Arabidopsis thaliana* by ion chromatography coupled to electrospray ionization tandem mass spectrometry (IC–ESI–MS–MS) was described. In the optimized method, over a dozen phosphorous compounds, including ADP, sugar phosphate (e.g., fructose 1,6-bisphosphate, galactose 1-phosphate, glucose 1-phosphate), and 3-phosphoglyceric acid, were determined. More recently, [73], developed an improved method using high-performance ion chromatography inductively coupled plasma mass spectrometry (HPIC-ICP-MS) by adapting strong anion exchange chromatography with acidic gradient elution for the simultaneous analysis of Pi and InsP6 in plant samples. Good selectivity and sensitivity, tolerance towards buffers and acids, and good quantification are undoubtedly pros of ICP-MS for substances containing phosphorus atoms.

High-performance liquid chromatography (HPLC) also found application in the separation of phytic acid in plant samples. The differential refractive index detector was the first detector to be used successfully in the HPLC analysis of different inositol phosphates. Since it has some drawbacks, such as being the least sensitive of the detectors, it can detect only the changes in the refractive index and must be used only in isocratic systems. The Burbano group [74] proposed a methodology for purification of legume extracts using a strong anion exchange column to separate InsP3 to InsP6 from lower inositol phosphates (InsP1 and InsP2) and analysis by ion-pair chromatography on a C18 reversed-phase column. Kwanyuen and Burton proposed a rapid and simple procedure for determining the phytate content in plant seeds (soybeans) that requires minimal sample preparation [75]. HPLC analysis can easily be set up for automation with commercially available equipment for a large number of samples. Harland with coworkers analysed phytate in 82 commonly consumed foods derived from plant seeds to evaluate the risk of zinc deficiency [76]. An ion-pair high-performance liquid chromatography method with refractive index detection has been developed on an analytical scale and as a preparative purification method to analyse phytates.

Independent of the determination of total phosphorus content, inorganic or organic phosphorus compounds were the aim of the presented efforts. All those attempts were destructive with respect to plants and dedicated to the examination of appropriate compounds or narrow classes of structurally related phosphorus forms. These aspects may be assumed to be an important weakness of phosphorus determination in plant matrices, although an increasing number of sophisticated techniques have provided increasing precision in the quantitative and qualitative determination of specific phosphorus derivatives. Therefore, the development of methods that somehow balance the examination of various P compounds and their quantification during the same run, simplify the sample pretreatment and guarantee the robustness of the determination process, is still highly desirable. The use of nuclear magnetic resonance spectroscopy, especially the ^31^P NMR technique, seems to be an inspiring result of these searches.

### Nuclear magnetic resonance spectroscopy as a tool for analysing plant metabolites

Currently, nuclear magnetic resonance spectroscopy (NMR spectroscopy) is a versatile technique that supplies information about the integration and regulation of metabolic pathways of plants through a combination of in vivo and in vitro measurements. This method, or rather specific techniques being developed, allows identification, quantification, and localization of metabolites, visualization of the intracellular environment, and exploration of pathways and their operation [77]. NMR spectroscopy exploits the magnetic properties of specific atomic nuclei, the nuclear spin, and can be used to determine the physical and chemical properties of atoms or the molecules in which they are contained. The most common nuclei exhibiting such magnetic properties and simultaneously used in experiments with plant samples are the highly abundant isotopes ^1^H (99.985% in nature) and fortunately ^31^P NMR (100.000% in nature) and the low abundance isotopes ^13^C (1.108% in nature) and ^15^ N (0.370% in nature). NMR signals (i.e., resonance) are observed when the sample located in a strong magnetic field is irradiated with pulses of radiofrequency electromagnetic radiation. Each nucleus within a molecule experiences a slightly different magnetic field because of its distinct chemical environment and thus absorbs energy at a slightly different frequency. NMR signals are characterized by their frequency (chemical shift), intensity, fine structure, and magnetic relaxation properties, all of which reflect the precise environment of the detected nucleus. This powerful and theoretically complex analytical method can provide detailed information about the structure, dynamics, reaction state, and chemical environment of molecules. Thus, NMR spectra often contain a wealth of information about the identity of the molecules in the sample, and it is on this basis that NMR can also be used to identify and quantify metabolites in samples of biological origin [78].

NMR offers an array of detection strategies that can be tailored to the type of plant sample and the metabolic problem that is being addressed. However, the nature of the NMR measurements required for these research tasks, particularly regarding the hardware requirements, the detection scheme, and the sensitivity of the analysis, is extremely variable [77].

Nevertheless, NMR spectroscopy is thought to be nondestructive, noninvasive, nonbiased, easily quantifiable, and requires few or no extra steps for sample preparation or fractionation. The resulting spectra can be recorded from cell suspensions, tissues, and even whole plants and from plant extracts or purified metabolites. NMR has become a mainstay for determining the structures of novel compounds [77, 79]. Additionally, NMR is very automatable, easy to perform, and highly reproducible, making high-efficiency, large-scale metabolomic studies much more practicable with NMR spectroscopy than with LC–MS or GC–MS [79]. NMR is a very suitable method to carry out metabolomic analysis in plants because it allows the simultaneous detection of diverse groups of abundant primary (e.g., sugars, amino acids, organic acids) and secondary (e.g., flavonoids, alkaloids, terpenoids) metabolites. This method enables the measurement of specific inorganic metabolites or ions and reflects the real molar levels of metabolites present in a plant [25, 79]. Another strong point of this technique is that researchers can record NMR spectra for multiple different nuclei, such as ^1^H, ^13^C, ^15^ N, and ^31^P, either separately or simultaneously to study different classes of metabolites. Furthermore, the correlation between two and even three different nuclei can be measured using multidimensional NMR techniques, which creates the possibility of analysing interactions between those substances. Nuclear magnetic resonance also supports metabolite imaging and metabolic analysis of living samples through magnetic resonance spectroscopy (MRS) and magnetic resonance imaging (MRI). As a result, NMR is ideal for real-time metabolite profiling of living cells, so real-time metabolic flux analysis can only be performed using NMR spectroscopy [79]. Nevertheless, NMR spectroscopy also has some disadvantages, the major of which is perhaps its low sensitivity compared to chromatographic separation coupled with any kind of mass spectrometry detection. Consequently, the amounts (e.g., volume, weight) of samples required for NMR-based analyses are larger than those required when using other analytical methods. However, the sensitivity of NMR spectroscopy has increased enormously because of recent improvements in NMR hardware [25].

In 1995, Roberts and Xia described one-dimensional NMR methods for the study of higher plants. Authors explain that NMR spectroscopy can provide information on the types of low-molecular-weight metabolites in plant cells, their relative concentrations, their mobility, and their interactions with other species such as H^+^ or paramagnetic ions [80]. Only a few years later, in 2001, the widespread use of NMR to analyse plant physiology, development and metabolism was discussed [77, 81]. A 2017 review by Deborde et al. illustrated how NMR spectroscopy, with its broad variety of experimental approaches, has contributed widely to the study of plant primary or specialized metabolism in very diverse ways. Authors presented recent developments of one-dimensional and multidimensional NMR methods to study various aspects of plant metabolism [82]. Currently, nuclear magnetic resonance is a powerful tool for metabolite profiling in higher plants [25, 78, 83, 84] and enables the dynamic investigation of plant metabolism that is virtually unmatched by any other analytical technique. NMR metabolomic analysis permits the identification of metabolites by comparing NMR data with references or by structure elucidation using two-dimensional experiments [25].

### Simultaneous determination of the chemical nature and amount of phosphorus components of plant tissues using ^31^P NMR techniques

The employment of ^31^P NMR spectroscopy has greatly improved the understanding of P species in plants, particularly the organic P forms quantified in relative terms. NMR is a powerful approach for plant metabolite profiling and provides a capacity for the dynamic exploration of plant metabolism that is virtually unmatched by any other analytical technique [81].

Because of 100.00% natural abundance, a spin of ½ and moderately large magnetic moment phosphorus is classified as an easily measurable NMR element; therefore,^31^P NMR provides a detailed characteristic of phosphorus-containing compounds with very high sensitivity to the chemical environment of the nucleus [85]. Moreover, those experiments and respective results are usually standardized because the chemical shift of the signals is referenced to an external 85% phosphoric acid. Signals (peaks) are defined by three parameters: chemical shift, line width, and peak height. The peak assignment is often based on literature data, which, together with the principles of ^31^P NMR, is well explained in review articles, textbooks, and methodological papers [86–88]. The main advantage of the ^31^P NMR technique is that all P species, including inorganic P forms and most of the organic forms, are visible in their respective spectra and therefore can be simultaneously characterized without the need for any complex cleaning and prefractionation procedures. These advantages are the reason that even high (in comparison to typical methods used for phosphorus quantification) detection limits and its vulnerability to the interferences resulting from the heterogeneous physical and chemical properties of the samples, as well as the natural association of P with paramagnetic ions such as iron and manganese, do not discriminate the use of phosphorus NMR [85]. One should also be aware that this technique can only detect phosphorus-containing molecules based on bond class, which makes this method not ideally suited to distinguish between inorganic polyphosphate and other molecules that also contain phosphoanhydride bonds, such as nucleotides. However, ^31^P NMR is typically applied to indicate the relative abundance of polyP in comparison to other P bond classes, such as pests and phosphonates, rather than to provide a direct measurement of the polyP concentration. Notably, ^31^P NMR measurements can be carried out with solid samples (solid-state NMR), in vivo or after extraction (liquid-state NMR), which provide simultaneous detection of various P metabolites, making this technique an extremely powerful and least disruptive tool for online detection for phosphometabolomic profiling under different experimental conditions [41].

#### Exemplary analyses of phosphorus compounds in plant materials using ^31^P NMR

In recent decades, ^31^P NMR spectroscopy has been widely used to simultaneously identify and quantify all phosphorus-containing compounds. This direct technique has been applied to in vivo studies of seeds and in vitro studies of the metabolism of P species in extracts of different parts of plants. Both variants provide detailed knowledge of the composition of the phosphorus compounds in individual parts of the plant and allow a detailed description of metabolic transformations. Moreover, in vivo ^31^P NMR studies of seeds have been used to determine the water content and intracellular pH during maturation and germination [89]. This information is well correlated with the first references to the use of ^31^P NMR in the analysis of plant material regarding the possibility of using this technique to distinguish the distribution of phosphorus between the vacuole and cytoplasm in higher plants due to changes in the environmental pH of the phosphate molecules. Moreover, the peak areas were proportional to the amount of phosphorus in a particular environment, enabling quantitative comparisons of the content of compartment Pi [90]. This technique permits the localization of phosphate in different subcellular compartments in various plant organs and homogenous plant culture cells [77]. In the late 1980s, phosphorus NMR provided valuable information about remobilization of this element on vacuolar inorganic phosphate pool size in soybean leaves with respect to phosphorus nutrition and plant development [90] and total mobile phosphorus content and nucleotide concentrations under different levels of acidity and mineral stresses [91]. Moreover, the latter authors demonstrated that corn root tips could be studied by ^31^P NMR for extended periods under both neutral and acidic conditions with little change in intracellular pH and distribution of observable phosphate-containing compounds. Generally, the extraction of corn roots by CaSO_4_ allows the determination of phosphorus compounds such as Pi vacuoles (Pv), Pi cytoplasms (Pc), ATP (γ, α, β), AMP, UDPG and NAD nucleotides, glucose-6-P, and fructose-6-P. In 1993, Crans and collaborators used ^31^P NMR spectroscopy to compare the content of phosphorus compounds in aqueous, acidic, and organic extracts of *Phaseolus vulgaris* seeds [89]. Usually, aqueous and acidic solvents are used to extract inorganic phosphate, sugar phosphates, and phytate, whereas treatments with organic solvents are used to extract phospholipids and nucleotides. In the Crans study, various ethanol extraction procedures were compared to two chloroform/methanol procedures and have been found to complicate the qualitative analysis of phospholipid profiles from dry cotyledons because of the numerous artefacts that were observed in the spectra. Other phosphorus compounds were extracted using perchloric acid, trichloroacetic acid, trichloroacetic acid in ether, hydrochloric acid, boiling water, and aqueous HEPES. Low concentrations of phosphosugars and other phosphorus metabolites were found in all aqueous and acidic extractions. High concentrations of phytate were found in all these extracts, with the difference that ^31^P NMR spectra of aqueous extractions did not show phytate resonance, attributed to the complexation of phytate. The spectra of aqueous extracts contained a broad resonance peak at 0.1 ppm, possibly wrongly assigned to protein-bound RNA. Bearing in mind that a plant cell contains more DNA than RNA, protein-bound nucleic acids seem to be a more accurate term. The authors concluded that exhaustive analysis of phosphorus metabolites in seeds requires a minimum of four extracts, which means an aqueous, acidic, nucleotide, and phospholipid extraction procedure. Although such a procedure is not overly complicated, efforts were made to simplify it by searching for a more universal solvent in the following years [89].

Currently, NaOH-EDTA is the most widely used extractant for ^31^P NMR spectroscopy for all parts and organs of plants. Noack et al. using P speciation by NMR, quantified various inorganic and organic P forms in the NaOH-EDTA extracts of stems, chaff and seeds collected from various crops (wheat, barley, oat, rye, canola, bean, lupin and pea) [92]. The main forms of P detected in the stem and chaff were orthophosphate, phospholipids and nucleic acids. Phytate was the dominant P species in seeds and constituted almost half of the total P in chaff but was only detected in minor amounts in stem residue. The majority of P in stems was water-extractable and was detected as orthophosphate [92]. It is well known that as plants approach maturity and start to senesce, the primary sink for phosphorus is the seeds, but it is unclear how the plant’s P status affects the resulting P concentration and speciation in the seeds and remaining plant parts. Also Noack with coworkers conducted a study in which they measured how P speciation in different parts of wheat and canola (root, stem, leaf, chaff/pod and seed) is affected by plant P status [17]. Moreover, the authors showed that phytate was the dominant form of P in seeds, whereas orthophosphate was the dominant form of P in other plant parts. Ebuele et al. investigated the P species in plants from a natural vegetation system dominated by brackens and bluebells [93]. The results indicate that all bracken (blade ˃ stipe ˃ rhizome) and bluebell parts (leaves ˃ scapes ˃ flowers ˃ roots ˃bulbs ˃ seeds) contained a significant percentage of Pi in the form of orthophosphate. Interestingly, myo-IP6 was the most abundant organic P form detected in bluebell bulbs and seeds but not in any other bluebell or all bracken parts. The other species detected in all bluebell plant parts and bracken stipes and blades were phospholipid degradation products (α- and β-glyp) and AMP. The other inorganic P species detected only in bluebell seeds was pyrophosphate. Other monoester and diester P forms were also present in most NaOH-EDTA plant extracts. In a continued study, cumin, fennel, flax, mustard, poppy, and sesame seeds were analysed for P species by the same group [94]. The results demonstrated that NaOH-EDTA extracts were similarly effective, providing data about the presence of orthophosphate monoesters (phytate, glycerophosphates, mononucleotides) and orthophosphate diesters. The obtained results suggest that P transferred from the plant vegetative parts (leaves, roots, stems, flowers) to the developing seeds during maturation is converted to organic P (phytate) in addition to being stored as inorganic P (orthophosphate) [94]. The next step was performed by Cai and coworkers, who combined ^31^P liquid and solid-state NMR spectroscopic methodology with a new extraction scheme and data analysis method to perform a quantitative investigation of phosphorous circulation in germinating sesame seeds in the dark and under illumination with and without the addition of a growth hormone [95]. They found that the metabolism of phosphorus was temperature-dependent and under the influence of illumination and hormones. Interestingly, illumination and hormones do not affect the final residual concentration of phytate. Moreover, phytate does not flow out of cotyledons, and the phosphorous flowing to other parts of the plant is always in inorganic form. The overall evolution profile of phytate consumption displays a Gaussian decaying trend. These findings can be explained with a dynamic model of phytate conversion. Nanganoa and collaborators performed studies in which they identified and quantified the various phosphorus species in leaf litter and crop residues from cocoa farms, oil palm, rubber, and banana plantations [34]. Orthophosphate monoesters were the second major P group detected by ^31^P NMR in all samples, with phytate detected only in palm male inflorescences. Orthophosphate diesters were detected only in fresh palm fronds, while pyrophosphate was detected in trace amounts in all samples except in fresh palm fronds. The use of various aspects of the ^31^P NMR technique allows us to create an impressive set of data regarding phosphorus species in plants. This information collected in Table 2 is primarily a comprehensive review of information about inorganic and organic forms of phosphorus present in organs and tissues of different plant species.

**Table 2:** Exemplary information on the application of ^31^P NMR technique in analysis of plant materials

Common name	Taxonomic name	Plant part	Form of phosphorus													Extraction method (extractant) Extracting solution	Citation
			OrthoP	PyroP	Phosphosugars	Phytate	Glycerolphosphates	Lysophosphatidylcholine	Phosphatidylinositol	Choline phosphate	Phosphatidylcholine	phosphorylated nucleotides	RNA	Other mono	Phosphodiester(s) (PE)		
Banana	*Musa sp.*	Fresh pseudostem, Decaying pseudostem	x	x										x		NaOH-EDTA	Nanganoa and Njukeng [38]
Barley	*Hordeum vulgare*	Stem	x	x		x	x						x			NaOH-EDTA	Noack et al. [92]
		Chaff	x			x	x						x				
		Seed	x			x	x										
Bean	*Phaseolus vulgaris L.*	Seeds cotyledons	x		x	x	x			x	x	x	x		x	Aqueous Hepes	Crans et al. [89]
			x		x	x	x				x	x	x		x	Boiling water	
					x	x		x	x		x				x	Ethanol	
			x		x	x	x									Trichloroacetic acid (TCA)	
			x		x	x	x			x						Trichloroacetic/ether (TCA/ether)	
			x		x	x	x			x						HCl	
			x		x	x	x			x						Perchloric acid (HClO_4_)	
									x	x					x	Chloroform–methanol A Chloroform–methanol B	
		Stem	x	x			x						x			NaOH-EDTA	Noack et al. [92]
		Chaff	x	x			x						x				
		Seed	x			x	x						x				
Bluebell	*Hyacinthoides non-scripta* (L.) Chouard ex. Rothm	Roots	x				x					x		x		NaOH-EDTA	Ebuele et al. [93]
		Bulbs	x			x	x					x					
		Seeds	x	x		x	x					x		x	x		
		Scapes															
		Leaves	x				x					x		x	x		
		Flowers															
Bracken	*Pteridium aquilinum* (L.) Kuhn	Rhizome	x											x		NaOH-EDTA	Ebuele et al. [93]
		Stipes	x				x					x		x			
		Blades	x				x					x		x	x		
	*Brassica napus*	Stem	x	x			x						x			NaOH-EDTA	Noack et al. [92]
		Chaff	x			x	x						x				
		Seed	x			x											
		Root	x			x	x					x				NaOH-EDTA	Noack et al. [17]
		Stem	x			x	x										
		Leaf	x				x										
		Pod	x	x		x	x										
		Seed	x			x	x					x					
Cocoa	*Theobroma cacao*	Pod husk, Senescent leaves (litter)	x	x										x		NaOH-EDTA	Nanganoa and Njukeng [38]
Corn	*Zea mays L.*	Root	x		x							x				CaSO_4_	Pfeffer et al. [91]
Cumin	*Cuminum cyminum*	Seed	x	x		x	x							x		NaOH-EDTA	Ebuele et al. [94]
Fennel	*Foeniculum vulgare*	Seed	x	x		x	x							x		NaOH-EDTA	Ebuele et al. [94]
Flax	*Linum usitatissimum*	Seed	x	x		x	x							x		NaOH-EDTA	Ebuele et al. [94]
Lupin	*Lupinus angustifolius*	Stem	x	x			x						x			NaOH-EDTA	Noack et al. [92]
		Chaff	x				x						x				
		Seed	x			x	x						x				
Mustard	*Sinapis alba* sp.	Seed	x	x		x	x							x	x	NaOH-EDTA	Ebuele et al. [94]
Oat	*Avena sativa*	Stem	x	x			x						x			NaOH-EDTA	Noack et al. [92]
		Chaff	x			x	x						x				
		Seed	x			x	x										
Palm	*–*	Fresh fronds	x											x	x	NaOH-EDTA	Nanganoa and Njukeng [38]
		Empty fruit bunches	x	x										x			
		Male inflorescence	x	x		x								x			
Pea	*Pisum sativum L.*	Stem	x	x			x						x			NaOH-EDTA	Noack et al. [92]
		Chaff	x				x						x				
		Seed	x			x	x						x				
Poppy	*Papaver sp*	Seed	x	x		x	x							x	x	NaOH-EDTA	Ebuele et al. [94]
Rubbertree	*Hevea brasiliensis*	Senescent leaves (litter)	x	x										x		NaOH-EDTA	Nanganoa and Njukeng [38]
Rye	*Secale cereale*	Stem	x	x			x						x			NaOH-EDTA	Noack et al. [92]
		Chaff	x				x						x				
		Seed	x			x	x										
Sesame	*Sesamum indicum*	Seed	x	x		x	x							x	x	NaOH-EDTA	Ebuele et al. [94]
		Seed	x			x										HClO_4_-EDTA	Cai et al. [95]
Soybean	*Glycine max*	Leaves			x							x				Buffer (Sorbitol/Hepes)	Lauer et al. [90]
Wheat	*Triticum aestivum*	Root	x	x		x	x					x				NaOH-EDTA	Noack et al. [17]
		Stem	x			x	x										
		Leaf	x				x										
		Chaff	x			x	x										
		Seed	x			x	x					x					
		Stem	x	x		x	x						x			NaOH-EDTA	Noack et al. [92]
		Chaff	x			x	x						x				
		Seed	x			x	x										

## Conclusions

From relatively simple colourimetric procedures based on chemical transformations with the creation of specific chromophores, through more sophisticated instrumental methods allowing the quantification of phosphorus in plants, to NMR, which combines the quantification and speciation of phosphorus forms, we can obtain valuable information about the phosphorus profile (phosphorome) of plants. There was a good correlation between searching for solving problems addressed to phosphorus determination and developing more advanced instrumental methods that allow it to be performed in a less costly and time-consuming manner. Many evaluation methods allow appropriate accuracy and repeatability when the matter of determination is the content of inorganic phosphorus or total phosphorus. It is possible to use simple colourimetric procedures (molybdenum blue colourimetric method, malachite green assay) and more advanced instrumental methods, e.g., FAAS, ETAAS, ICP-OES, ICP-AES or ICP-MS, IC (Table 1). Based on our laboratory experience and according to the systematic review presented hereby, it may be concluded that each analytical problem related to the determination of phosphorus requires an individual approach and should be dedicated to a defined problem. However, when the form of phosphorus or knowledge about the dynamics of the transformation of this element in the body of living beings is the point, nuclear magnetic spectroscopy, especially the variants of the ^31^P NMR technique, is much more suitable, even though the limit of detection (LOD) of this method is far higher than that obtained in other methods. Nevertheless, the most promising approach seems to be a holistic approach to the determination of phosphorus in plant tissues and organs: the combination of phosphorus quantification and profiling, tiding different methods and techniques with respect to the goal of determination. Such an approach provides the unique opportunity to develop phosphoromics as the new aim of analytical challenges.

## Data Availability

Not applicable.

## Abbreviations

AEC: Anion exchange chromatographic
ETAAS: Electrothermal atomic absorption spectrometry
ETV: Electrothermal vaporization
FAAS: Flame atomic absorption spectrometry
GC–MS: Gas chromatography–mass spectrometry
HPAEC: High-performance anion-exchange chromatography
HPIC: High-performance ion chromatography
HPLC: High-performance liquid chromatography
HR-ETAAS: High-resolution continuum source electrothermal atomic absorption spectrometry
IPC: Ion pair chromatography high-performance ion chromatography (HPIC)
IC: Ion chromatography
ICP-AES/ICP-OES: Inductively coupled plasma atomic emission spectrometry or inductively coupled plasma optical emission spectrometry
ICP-MS: Inductively coupled plasma mass spectrometry
InsP6: Hexaphosphate inositol, phytic acid
LC–MS: Liquid chromatography–mass spectrometry
LOD: Limit of detection
LS-FAAS: Line source flame atomic absorption spectrometry
MRI: Magnetic resonance imaging
MRS: magnetic resonance spectroscopy
NMR: Nuclear magnetic resonance spectroscopy
Pi: Orthophosphate group
Po: Organic phosphorus
polyP: Polyphosphate
PPhy: Phytate phosphorus
SIMS: Secondary ion mass spectrometry
T6P: Trehalose-6-phosphate
TEM: Transmission electron microscope
TP: Total phosphorus
XRF: X-ray fluorescence
